# Impacts of alpine wetland degradation on the composition, diversity and trophic structure of soil nematodes on the Qinghai-Tibetan Plateau

**DOI:** 10.1038/s41598-017-00805-5

**Published:** 2017-04-12

**Authors:** Pengfei Wu, Hongzhi Zhang, Liwei Cui, Kyle Wickings, Shenglei Fu, Changting Wang

**Affiliations:** 1 College of Life Science and Technology, Southwest University for Nationalities, Chengdu, 610041 China; 2 Department of Entomology, New York State Agricultural and Experiment Station, Cornell University, Geneva, NY 14456 USA; 3 School of Environment and Planning, Henan University, Kaifeng, 475004 China

## Abstract

Alpine wetlands on the Qinghai-Tibetan Plateau are undergoing degradation. However, little is known regarding the response of soil nematodes to this degradation. We conducted investigations in a wet meadow (WM), a grassland meadow (GM), a moderately degraded meadow (MDM) and a severely degraded meadow (SDM) from April to October 2011. The nematode community taxonomic composition was similar in the WM, GM and MDM and differed from that in the SDM. The abundance declined significantly from the WM to the SDM. The taxonomic richness and Shannon index were comparable between the WM and MDM but were significantly lower in the SDM, and the Pielou evenness showed the opposite pattern. The composition, abundance and diversity in the WM and SDM were relatively stable over time compared with other habitats. The abundances of all trophic groups, aside from predators, decreased with degradation. The relative abundances of herbivores, bacterivores, predators and fungivores were stable, while those of omnivores and algivores responded negatively to degradation. Changes in the nematode community were mainly driven by plant species richness and soil available N. Our results demonstrate that alpine wetland degradation significantly affects the soil nematode communities, suppressing but not shifting the main energy pathways through the soil nematode communities.

## Introduction

Wetlands are important ecosystems because of the wide range of services they perform, such as supporting biodiversity, carbon storage, and water regulation and purification^1,2^. However, wetlands have been identified as being at risk from climate change through higher temperatures, greater evapotranspiration and altered precipitation patterns that modify hydrological regimes^3,4^. Among the ecosystems on the Qinghai-Tibetan Plateau, wetland is one of the largest, occupying 4.9 × 10^4^ km^2^ ^5^. Located on the eastern edge of the Qinghai-Tibetan Plateau, the Zoigê Wetland is the world largest marsh, with an area of 1.2 × 10^4^ km^2^ ^6^.

During the period from 1955–1996, the mean annual temperature across the Qinghai-Tibetan Plateau increased at a rate of 0.16 °C per decade, which exceeded that in the Northern Hemisphere and the corresponding latitudinal zone over the same period^7^. The increasing temperature has caused the alpine wetlands to become drier and drier. Grazing intensity on the Qinghai-Tibetan Plateau increased from 82.3 × 10^4^ sheep ha^−1^ year^−1^ in the 1950s to 306.7 × 10^4^ sheep ha^−1^ year^−1^ in 2005, i.e., 64.4% higher than the theoretical grazing capacity of this ecosystem^6^. Increased temperature^8^ and intensified livestock grazing^6,9^ have caused a degradation cross the wetlands on the Qinghai-Tibetan Plateau. Particularly for the Zoigê Wetland, this resulted in drier conditions and a shift from wet meadows to grassland meadows, followed by moderately degraded meadows and ultimately sandy meadows at severely deteriorating sites^10^. The plant species composition differs across this degradation gradient, and the ecotype of the dominant species shifts from humidogenes and aquatics to mesophytes and xerophytes^11^. At severely degraded sites, reduced plant biomass and organic C input to soil have resulted in the loss of the soil organic layer and subsequent exposure of the underlying sandy loam^12^. Soil nutrient contents have also declined with degradation^13^. The soil fauna of alpine wetlands are likely also impacted by degradation; however, only a few studies have addressed this to date^14^.

Nematodes represent a major component of the soil microfauna^15^, occupy key positions at most trophic levels in the soil food web, and modify many ecosystem processes, including succession, material cycling, and energy flow^4,16,17^. However, they are sensitive to environmental factors and are therefore widely used as ecological indicators of soil conditions^18,19^. The composition, abundance, trophic structure, and diversity of a nematode community can be affected by plant species identity, species composition, diversity and functional groups^16,20–24^. Moreover, dynamics of soil organic matter content^25–27^, nitrogen^28–31^, phosphorus^32,33^, temperature^34^ and moisture^35^, texture and salinity^36^ have significant effects on nematode abundance, diversity and trophic structure. The taxon composition, diversity and trophic structure of soil nematode communities are likely to be affected by plant community and soil properties associated with alpine wetland degradation. However, soil nematode diversity in alpine wetlands has rarely been studied, and the changes that occur in soil nematodes during alpine wetland degradation are unknown. Therefore, investigating the responses of soil nematodes to alpine wetland degradation is crucial for understanding soil food webs and the ecological functions of this sensitive ecosystem.

The objectives of this study were to characterize the taxonomic composition of alpine wetland nematode communities and to quantify the impacts of alpine wetland degradation on soil nematode diversity and trophic structure. We hypothesized that in response to the degradation of alpine wetland, (1) the abundance and diversity of the soil nematode communities will decrease, and (2) the main energy flow pathways through the nematode communities will shift.

## Materials and Methods

### Site description

**Figure 1:**
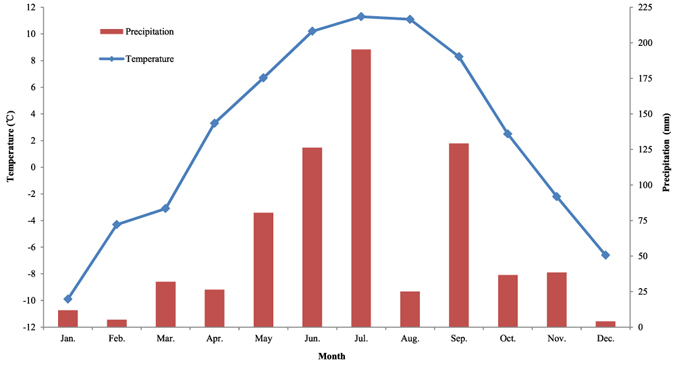
Total precipitation (mm) and monthly mean temperature (°C) at the study area in 2011. The data are from the Zoigê climate station.

The research site is located in the Zoigê Wetland (N 32°56′-34°19′, E 102°08′-103°39′), which includes Zoigê, Hongyuan and Aba Counties in Sichuan Province and Maqu and Luqu Counties in Gansu Province. The Zoigê Wetland lies in a transitional zone between semi-humid alpine and semi-humid temperate climates. The elevation ranges from 3,400 to 3,800 m. The mean annual temperature is 1 °C. The lowest mean monthly temperature occurs in January (−10.6 °C), while the highest is in July (10.8 °C). Annual precipitation ranges from 600 to 800 mm, and 86% of the precipitation occurs between late April and mid-October. The temperature and precipitation during the study period (2011) are shown in Fig. 1. The alpine wetlands in this region cover 6,180 km^2^, which is 31.5% of the entire Zoigê Plateau^37^. Currently, this area includes four habitats with different degradation status: wet meadow (WM), grassland meadow (GM), moderately degraded meadow (MDM), and severely degraded meadow (SDM) (Supplementary materials, Figures S1, S2, S3 and S4). The species composition of the plant communities and the soil texture of each habitat are provided in Table 1. The soil texture is peat soil for wet meadow, sandy loam for both grassland meadow and moderately degraded meadow, and sandy soil for severely degraded meadow.

**Table 1:** The main plant species and soil textures of the four habitats in Zoigê Wetland.

Habitats	Main plant species	Soil texture
WM	*Carex muliensis* Hand.-Mazz., *C. lasiocarpa* Ehrh., *C. meyeriana* Kunth., *Polygonum viviparum* L., *Taraxacum lugubre* Dahlst.*, Aster alpinus* L.*, Trollius ranunculoides* Hemsl. and *Geranium orientalitibeticum* R. Knuth.	Peat soil
GM	*Kobresia tibetica* Maxim.*, K. kansuensis* Kukenth., *Festuca airoides* Lam.*, Ranunculus tanguticus* (Maxim.) Ovcz.*, Carum carvi* L.*, Anaphalis lactea* Maxim.*, Anemone prattii* Hand.-Mazz.*, Elymus nutans* Griseb.*, Poa pratensis* L.*, T. lugubre*, and *Oxytropis kansuensis* Bunge	Sandy loam
MDM	*Potentilla anserina* L.*, K. tibetica, Ligularia virgaurea* (Maxim.) Mattf.*, Stellera chamaejasme* Linn.*, Leontopodium chuii* Hand.-Mazz.*, T. lugubre*, *R. tanguticus*, and *O. kansuensis*.	Sandy loam
SDM	*E. nutans* and *C. carvi*.	Sandy soil

WM: Wet meadow; GM: Grassland meadow; MDM: Moderately degraded meadow; SDM: Severely degraded meadow.

### Experimental design

Six plots (20 m × 20 m) one kilometer apart were marked with permanent signs and established in each of the four habitats. During April (winter), May (spring), July (summer) and October (autumn) in 2011, three soil samples (each approximately 500 g) were randomly collected from the 0–15 cm layer at 5 m intervals within each plot. Thus, a total of 288 soil samples (4 habitats × 6 plots × 3 samples × 4 seasons) were examined during the study period.

### Soil nematode extraction

In the laboratory, soil nematodes were extracted from 50 g of fresh soil from each sample using Baermann funnels. The gravimetric moisture content of the soil was determined so that response variables could be expressed on a dry weight basis. The extracted nematodes were preserved using 5% formalin and were then killed and fixed by the addition of boiling double-strength F.A. 4:1 (100 ml of 40% formaldehyde, 10 ml of glacial acetic acid, and 390 ml of distilled water)^26^. All nematodes in each sample were counted under 40× magnification using a stereomicroscope. The first 100 nematodes encountered in each sample were identified to the genus level under 400× magnification using a compound microscope (Leica DM4000B) according to the reference “*Pictorial keys to soil animals of China*”^38^. The nematodes were classified into six trophic groups, herbivores, bacterivores, predators, omnivores, fungivores and algivores, according to the references^39–41^.

### Estimation of plant and soil parameters

The environmental characteristics of the four habitats were investigated in April, May, July and October 2011. The species richness and coverage of the plant communities were measured within 1 m × 1 m sampling areas. Three replicate samples were established in each plot. The vegetation height was measured using a ruler with units of 1 cm. The vegetation coverage was measured using visual estimations in the field. The above- and below-ground biomass of each sample was harvested and dried to a constant weight at 80 °C in the laboratory. Soil samples (consisting of three replicates) were collected in each plot at a depth of 0–15 cm using a flat shovel. The soil samples were air-dried and passed through 2.00 and 0.25 mm sieves for chemical analyses. The soil chemical properties were determined according to well-established methods^42^. Specifically, the soil organic matter (SOM) content was determined using the Walkley-Black method. Total N was measured using the semi-micro Kjeldahl method, and plant-available N was determined using a micro-diffusion technique following alkaline hydrolysis. Total P was determined colorimetrically after wet digestion with sulfuric and perchloric acid, and available P was determined using the Olsen method. Total K was determined using a flame photometer, and available K was measured in 1 mol L^−1^ NH_4_OAc extracts using flame photometry. The soil bulk density content in the 0–15 cm layer was investigated using 200 cm^3^ soil cores (height: 52 mm; radius: 35 mm). The gravimetric soil moisture content was measured for each season using a ratio of the mass loss to the total dry mass of the soil samples after heating to a constant weight at 105 °C.

### Data analysis

First, the nematodes from three soil cores obtained from the same plot and sampling month were pooled as one sample. The abundance (the number of individuals per 100 g dry soil) and generic richness (mean number of genera per sample) were used to measure the response of the soil community to changes in habitats and seasons. Relative diversity indices, i.e., the Shannon index (${H}^{\text{'}}=-\sum _{i=1}^{s}{p}_{i}{\rm{i}}\,\mathrm{ln}\,{p}_{i}$) and the Pielou index (*J* = *H*′/ln*s*), were calculated at the genus level to evaluate the responses of diversity and evenness to changes in habitats and seasons^43,44^. To evaluate the changes in the trophic structure of the soil nematode communities, the abundances (individuals per 100 g dry soil) of the six trophic groups in the same sampling plot were calculated. The relative abundance (individual percentages) of each trophic group was also used to reveal the changes in trophic structure, given that the relative abundances, rather than abundances of different trophic groups, can directly reflect their relative importance in communities in some cases. Repeated-measures ANOVAs were performed using IBM SPSS 22.0 for Windows to evaluate the effects of the habitats (WM, GM, MDM and SDM), sampling months (April, May, July and October) and their interactions on the diversity indices and abundances of the nematode communities, abundances and relative abundances of trophic groups of soil nematodes.

Principal components analysis (PCA) was performed using Canoco for Windows 4.5 to evaluate the effects of habitats and sampling months on the composition of the soil nematode communities^45^. The PCA was run separately for each season, as well as for each habitat, to simplify data presentation. To reduce the number of variables and the figure complexity, these analyses were performed at the family level. The abundance data (ind. 100 g^−1^ dry soil) of each plot were log transformed before they were subjected to PCA. One-way ANOVA was used to evaluate the significant differences in the sample scores of the first two canonical axes (PC1 and PC2) among habitats (IBM SPSS 22.0 for Windows).

Additionally, the sample scores of the first two canonical axes (PC1 and PC2) of the communities, determined during April, May, July and October, were averaged across each of the six plots within each habitat. The same calculations were conducted on the abundance, generic richness, Shannon index and Pielou index of each community and on the abundances and relative abundances of the six trophic groups. Finally, stepwise multiple regression analysis was conducted to test the relationships between the soil nematodes and environmental parameters (IBM SPSS 22.0 for Windows).

## Results

### Plant communities and soil properties

Plant species richness and coverage were significantly lower in the SDM than in the WM, and the vegetation height and above-ground, below-ground and total biomass varied significantly among the four habitats (Table 2). The plant variables in the SDM were significantly smaller than those in other habitats (Table 2). The soil bulk density and pH increased, while the water content decreased significantly from the WM to SDM (Table 2). The contents of SOM and of total and available soil N, P and K varied significantly among the habitats, with the lowest values occurring in the SDM. In addition, the soil texture differed among the four habitats. For example, peat soil was found in the WM, sandy loam was found in the GM and MDM, and sandy soil in the SDM (Table 1).

**Table 2:** Mean values (mean ± S.E.) and significance tests of the environmental characteristics at different degradation phases of Zoigê Wetland (n = 6).

	WM	GM	MDM	SDM	*F*-value	*P*-value
**Plant community**						
Species richness	10.67 ± 1.21^a^	9.17 ± 1.17^a^	7.33 ± 0.82^b^	1.50 ± 0.55^c^	101.87	**<0.001**
Coverage %	92.33 ± 1.02^a^	98 ± 0.37^a^	70.67 ± 4.49^b^	6.00 ± 0.58^c^	328.09	**<0.001**
Height cm	31.33 ± 2.01^a^	40.33 ± 1.28^b^	20.5 ± 2.08^c^	8.5 ± 0.99^d^	68.87	**<0.001**
Aboveground biomass, g m^−2^	245.78 ± 25.29^a^	497.51 ± 49.56^b^	222.83 ± 17.36^a^	98.08 ± 12.75^c^	39.74	**<0.001**
Belowground biomass, g m^−2^	1812.12 ± 224.22^a^	3506.69 ± 622.57^a^	436.66 ± 103.88^b^	164.06 ± 33.51^c^	45.11	**<0.001**
Total biomass, g m^−2^	2057.91 ± 229.21^a^	4004.21 ± 619.89^b^	659.49 ± 99.57^c^	262.14 ± 41.49^d^	60.80	**<0.001**
**Soil properties**						
Bulk density, g cm^−3^	0.47 ± 0.02^a^	0.92 ± 0.03^b^	0.88 ± 0.03^b^	1.36 ± 0.06^c^	90.96	**<0.001**
Water content, %	77.97 ± 3.63^a^	32.43 ± 3.48^b^	37.27 ± 2.35^b^	4.8 ± 0.29^c^	117.76	**<0.001**
Organic material, g kg^−1^	144.49 ± 3.42^a^	69.5 ± 3.41^b^	102.88 ± 3.05^c^	4.39 ± 0.53^d^	426.29	**<0.001**
Total N, g kg^−1^	6.23 ± 0.21^a^	3.58 ± 0.13^b^	4.46 ± 0.17^c^	0.29 ± 0.03^d^	277.82	**<0.001**
Available N, mg kg^−1^	181.68 ± 2.61^a^	98.74 ± 3.08^b^	112.89 ± 3.09^c^	22.87 ± 1.66^d^	592.59	**<0.001**
Total P, g kg^−1^	1.07 ± 0.04^ab^	1.17 ± 0.03^a^	1.04 ± 0.02^b^	0.41 ± 0.01^c^	152.35	**<0.001**
Available P, mg kg^−1^	8.6 ± 0.63^a^	7.07 ± 0.50^a^	21.3 ± 2.27^b^	2.99 ± 0.09^c^	86.19	**<0.001**
Total K, g kg^−1^	14.38 ± 0.07^a^	18.59 ± 0.14^b^	18.12 ± 0.16^b^	11.81 ± 0.25^c^	362.03	**<0.001**
Available K, mg kg^−1^	152.24 ± 24.64^a^	369.69 ± 64.33^b^	501.35 ± 6.05^b^	35.48 ± 0.87^a^	36.86	**<0.001**
pH	6.11 ± 0.10^a^	5.95 ± 0.09^a^	7.06 ± 0.06^b^	8.46 ± 0.01^c^	240.47	**<0.001**

Statistically significant (*P* < 0.05) results from one-way ANOVA followed by Tukey’s multiple comparison test are shown in bold. Different superscript lowercase letters in the same row indicate significant differences at the *P* < 0.05 level.

### Soil nematode composition

A total of 78 nematode genera were identified across all samples, belonging to 38 families and 8 orders (Supplementary materials Table S1). Among the four habitats, the number of genera ranged from 51 to 65, and abundance ranged from 687.43 to 6826.94 ind. 100 g^−1^ dry soil. Overall, *Acrobeloides* and *Aphelenchus* were the dominant genera, accounting for 10.80% and 10.06%, respectively, of the total individuals collected. Tylenchida, Rhabditida and Dorylaimida were the three most abundant orders and represented 34.30%, 24.80% and 21.76%, respectively, of the total soil nematodes collected. Regarding the trophic groups, the percentages of bacterivores, herbivores, omnivores, fungivores and predators were, respectively, 32.10%, 20.90%, 17.60%, 15.73% and 12.62%, with algivores (1.05%) constituting the least abundant group (Table S1).

### Nematode community structure

**Figure 2:**
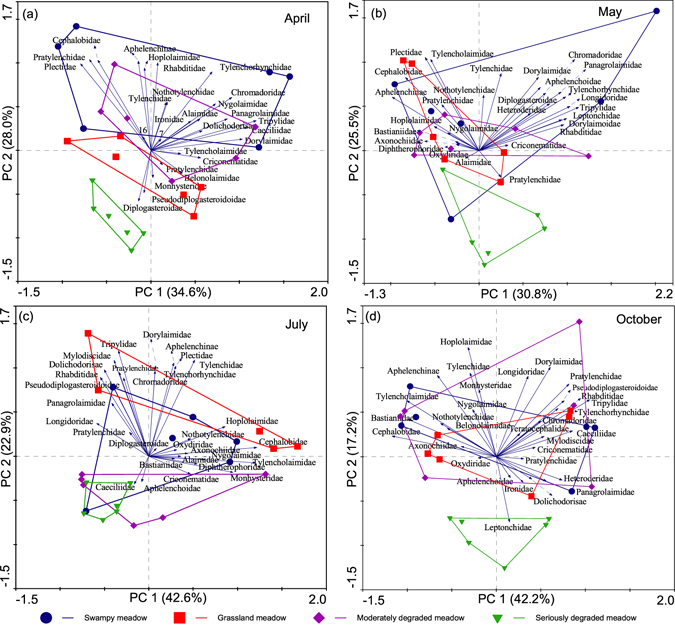
Variations in community structure of the soil nematodes in different Zoigê Wetland habitats in four months. Short arrows that indicate rare taxonomic groups were omitted for clarity.

The PCA results showed that the composition of the soil nematode communities varied among the four habitats (Fig. 2a–d). The nematode communities from the SDM separated clearly from the other habitats according to PC1 and PC2 in April, May and October (Fig. 2a,b and d); however, the SDM nematode communities overlapped with those of the WM and MDM in July (Fig. 2c). The one-way ANOVA results showed that only the PC2 factor scores differed significantly among habitats in each month (April: *F* = 15.34, *P* < 0.001; May: *F* = 8.02, *P* < 0.001; July: *F* = 15.02, *P* < 0.01; October: *F* = 10.44, *P* < 0.001). On the whole, the main taxonomic groups associated with the separation of PC1 and PC2 across the sampling month were Cephalobidae, Tylencholaimidae, Aphelenchinae, Dorylaimidae, Tripilidae and Plectidae, but the pattern varied with sampling month (Fig. 2a–d).

**Figure 3:**
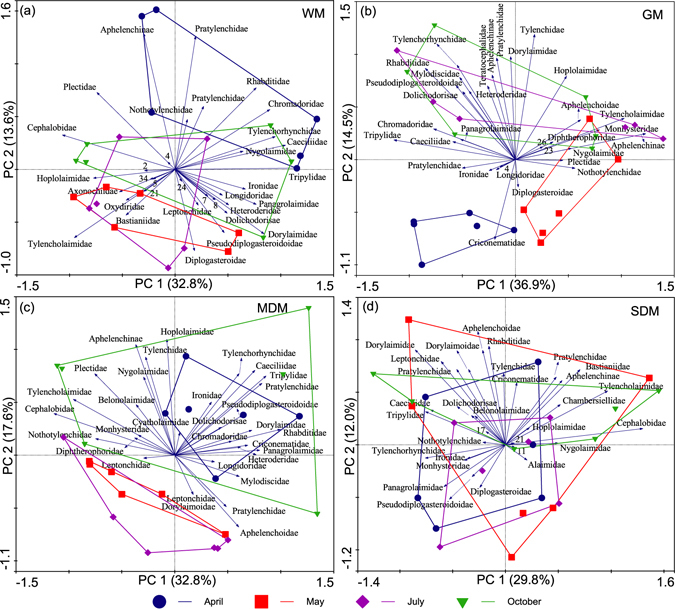
Seasonal variations in community structure of the soil nematodes of Zoigê Wetland. Short arrows indicating rare taxonomic groups were omitted for clarity.

The composition of the soil nematode communities also varied between sampling months, but the patterns differed among the habitats (Fig. 3a–d). For the GM, the communities in April were separated clearly from those in May, July and October by PC1 and PC2 (Fig. 3b), and the nematode communities in April and October were separated from those in May and July for the MDM (Fig. 3c). In contrast, the nematode communities differed little among sampling months in the WM and SDM (Fig. 3a and d). The significant differences among sampling months were only observed in the second axis factor scores for the WM (*F* = 6.81, *P* < 0.01), GM (*F* = 16.47, *P* < 0.001) and MDM (*F* = 9.24, *P* < 0.001). Additionally, the taxonomic groups determining the temporal differences of the communities varied among habitats (Fig. 3a–d).

### Nematode community abundance and diversity

**Figure 4:**
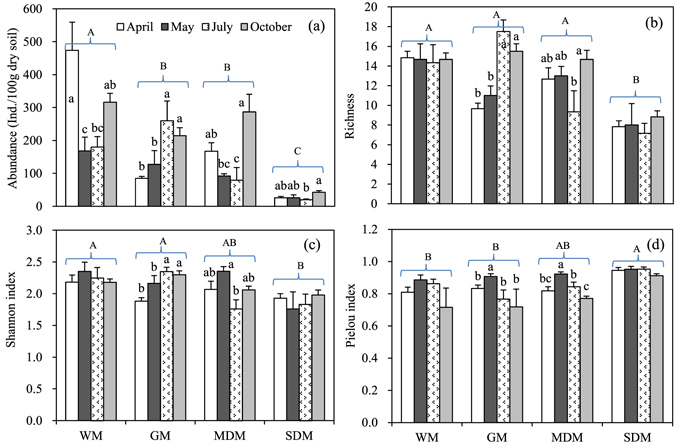
Spatio-temporal variations in abundance (**a**), richness (**b**), Shannon diversity (**c**) and Pielou index (**d**) of the soil nematode communities (mean + SE). Lower-case letters indicate temporal differences within habitats at the *P* < 0.05 level.

The abundance of nematodes decreased significantly from the WM to SDM (*P* < 0.001) and varied significantly among sampling months (*P* < 0.001) (Fig. 4a, Table 3). Nematode abundance also responded significantly to the interaction effects of habitat and sampling month (*P* < 0.01) (Table 3).

**Table 3:** Repeated-measures ANOVA results for the effects of habitats, sampling months and their interactions on the abundance and diversity of the soil nematode community.

Effects	df	Abundance		Taxonomic richness		Shannon index		Pielou index	
		*F*	*P*	*F*	*P*	*F*	*P*	*F*	*P*
Habitats	3, 20	107.41	**<0.001**	24.12	**<0.001**	10.60	**<0.001**	4.02	**0.022**
Sampling months	3, 60	10.65	**<0.001**	5.38	**0.008**	1.59	0.226	6.89	**0.003**
Habitats × Sampling months	9, 60	3.58	**0.001**	2.36	**0.024**	1.99	0.056	1.35	0.233

Statistically significant (*P* < 0.05) results are shown in boldface (n = 24).

The taxonomic richness, Shannon index and Pielou index differed between the WM and MDM, and the SDM showed significantly lower values for taxonomic richness (*P* < 0.001) and the Shannon index (*P* < 0.001) and a higher value for the Pielou index (*P* < 0.05) (Fig. 4b–d, Table 3). The taxonomic richness also responded significantly to sampling month (*P* < 0.01) and the interaction effects of sampling month and habitat (*P* < 0.05), with the Pielou index showing significant differences by sampling month (*P* < 0.01) (Table 3). However, the temporal patterns varied among the habitats, and significant temporal dynamics in diversity were only recorded for the GM and MDM (*P* < 0.05) (Fig. 4b–d).

### Nematode community trophic structure

**Figure 5:**
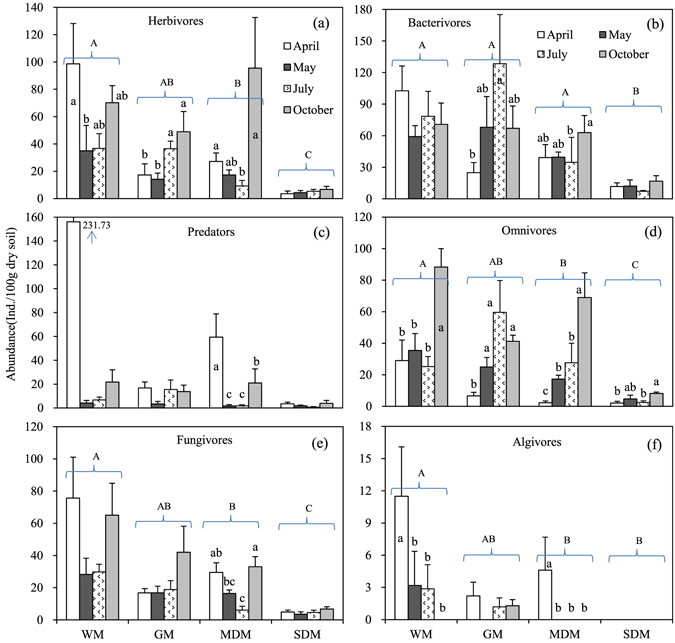
Spatio-temporal variations of the abundances of the six trophic groups across the four habitats (mean + SE). Lower-case letters indicate temporal differences within habitats at the *P* < 0.05 level.

**Figure 6:**
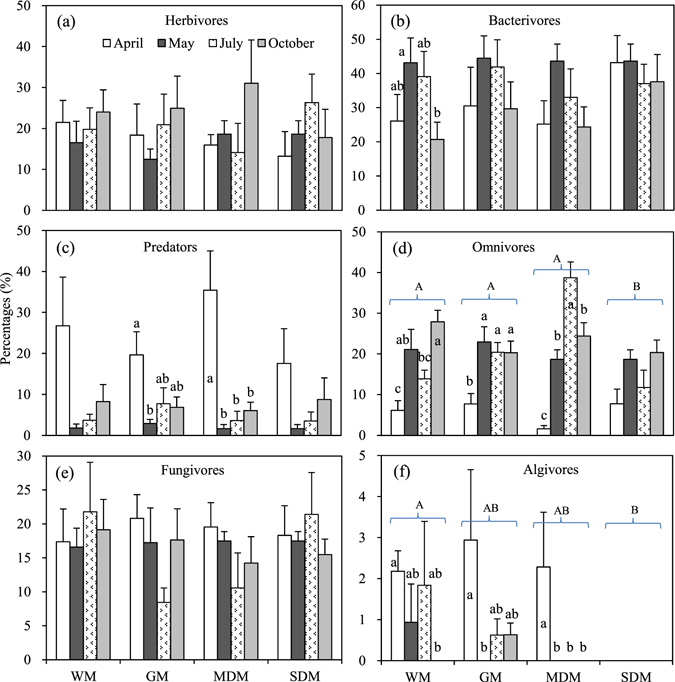
Spatio-temporal variations of the relative abundances of the six trophic groups across the four habitats (mean + SE). Lower-case letters indicate temporal differences within habitats at the *P* < 0.05 level.

With the exception of predators, the abundances of all trophic groups decreased significantly with increasing degradation (*P* < 0.001 or 0.01), with algivores disappearing from the SDM (Fig. 5a–f, Table 4). The abundances of all trophic groups, except bacterivores, varied significantly among sampling months (*P* < 0.001 or 0.01) (Table 4). The abundances of the herbivores, bacterivores and algivores were also sensitive to the interaction effects of habitat and sampling month (*P* < 0.001 or 0.05) (Table 4). The temporal patterns of individual trophic groups also differed among habitats (Fig. 5a–f). The relative abundances (individual percentages) of the omnivores and algivores declined significantly with habitats degradation (*P* < 0.001 or 0.05) (Fig. 6a–f, Table 4). Additionally, the temporal effects on the relative abundances were significant for the bacterivores, predators, omnivores and algivores (*P* < 0.001 or 0.01) (Fig. 6a–f, Table 4), and the temporal pattern of each trophic group differed among habitats (Fig. 6a–f).

**Table 4:** Repeated-measures ANOVA results for the effects of habitats, sampling months and their interactions on the abundances and relative abundances of trophic groups of the soil nematode communities.

Effects	df	Herbivores		Bacterivores		Predators		Omnivores		Fungivores		Algivores	
		*F*	*P*	*F*	*P*	*F*	*P*	*F*	*P*	*F*	*P*	*F*	*P*
**Abundance**													
Habitats	3, 20	33.74	**<0.001**	10.23	**<0.001**	3.05	0.053	42.30	**<0.001**	30.19	**<0.001**	6.19	**0.004**
Sampling months	3, 60	5.00	**0.004**	1.43	0.242	11.06	**<0.001**	35.44	**<0.001**	4.66	**0.005**	8.11	**<0.001**
Habitats × Sampling months	9, 60	2.22	**0.033**	3.36	**0.002**	1.68	0.115	1.86	0.077	1.10	0.375	2.45	**0. 019**
**Relative abundance**													
Habitats	3, 20	0.64	0.596	0.52	0.676	0.19	0.899	7.24	**0.002**	0.70	0.565	3.92	**0.024**
Sampling months	3, 60	0.85	0.470	8.20	**<0.001**	10.86	**<0.001**	22.63	**<0.001**	0.84	0.475	5.33	**0.008**
Habitats × Sampling months	9, 60	1.13	0.357	1.28	0.266	0.93	0.510	2.77	**0.009**	1.11	0.371	1.96	0.061

The statistically significant (*P* < 0.05) results are shown in boldface (n = 24).

### Impacts of environmental factors on soil nematodes

The results from the multiple regression analyses (Table 5) show that PC1 and PC2 were significantly correlated with average plant height (*P* < 0.05) and plant species richness (*P* < 0.001), respectively. Nematode abundance was correlated with plant species richness (*P* < 0.05) and soil available N (*P* < 0.01), while the Shannon index was negatively correlated with aboveground biomass (*P* < 0.05) and pH (*P* < 0.001). The taxonomic richness was found to correlate with plant species richness (*P* < 0.001) and the Pielou index to coverage (*P* < 0.001), respectively. The abundances of the bacterivores and omnivores were significantly correlated with plant species richness (*P* < 0.001), as were those of the fungivores and algivores to the soil water content (*P* < 0.001). In addition, the herbivore and predator abundances were significantly positively correlated with soil available N and total N contents (*P* < 0.001 or 0.01) (Table 5). Regarding the relative abundances of the six trophic groups, only omnivores and algivores were correlated with plant coverage and available N (*P* < 0.001 or 0.05) (Table 5).

**Table 5:** The partial correlation coefficients of multiple regression analyses (stepwise procedure) between soil nematode communities and environmental factors (n = 24).

		Plant species richness	Coverage	Height	Aboveground biomass	Water content	Total N	Available N	pH
Community index	PC1			0.25^*^					
	PC2	0.91^***^							
	Abundance	0.44^*^						0.59^**^	
	Richness	0.89^***^							
	Shannon				−0.43^*^				−0.79^***^
	Pielou		−0.66^***^						
Abundance of trophic groups	Herbivores							0.78^***^	
	Bacterivores	0.65^***^							
	Predators						0.54^**^		
	Omnivores	0.87^***^							
	Fungivores					0.83^***^			
	Algivores					0.60^***^			
Relative abundance of trophic groups	Herbivores								
	Bacterivores								
	Predators								
	Omnivores							0.61^***^	
	Fungivores								
	Algivores		0.49^*^						

The superscript stars ***, ** and * indicate significant correlations at the 0.001, 0.01 and 0.05 levels, respectively.

## Discussion

### Changes in soil nematode community composition and diversity

The soil nematode communities in the WM, GM and MDM were relatively similar, but they differed remarkably from that in the SDM. Soil nematode community patterns among habitats varied substantially with sampling months. These indicated that the compositions of soil nematode communities change in response to alpine wetland degradation, while further demonstrating that the impacts of wetland degradation are temporally variable. The observed shifts in the nematode communities may reflect differences in plant communities among habitats. Our analysis also shows that the community structure of the soil nematodes was influenced by plant species richness, which changed markedly in the MDM and SDM. Nematode communities are significantly different among vegetation types^46^, and plant species composition is one of the principal factors structuring soil nematode communities^24,47^. This relationship may result from the fact that increased plant diversity generally provides a variety of foods and habitats for soil invertebrates^48^. Apart from the effects of the plants on the soil nematode communities, the composition of soil invertebrate communities can also be affected by soil properties^49,50^. In the Zoigê Wetland, the soil parameters measured, including soil texture and moisture, differed significantly among the four habitats, and these differences were distinct between the MDM and SDM. Therefore, the degradations in the soil properties among the habitats might also be an important determinant of the taxonomic composition of soil nematodes in the alpine wetland ecosystem.

Our results suggest that the patterns in nematode abundance and diversity among habitats may be related to plant community and soil traits. Soil nematodes can be affected by changes in soil P, N and organic matter contents^25,30,32^. In our research, the decrease in plant species richness and increase in soil pH during the degradation progress would negatively affect soil nematode diversity according to the relationships between these variables. The plant community simplification can lead to the disappearance of some nematode species^51^. The Pielou index increased gradually with the degradation and was negatively correlated with plant coverage. Other researchers have also reported that the evenness of soil nematode communities was affected by shifts in plant community traits^15^. The increase of the evenness index suggests that competitive exclusion among different nematode taxa may decrease with alpine wetland degradation. Overall, the effects of plant communities and soil properties on the abundance and diversity of the soil nematodes indicate that the abundance and diversity of soil nematodes are more easily influenced by variations in plant communities than soil properties in an alpine wetland. However, compared with the GM, the taxonomic richness and Shannon index decreased only slightly in the MDM and decreased significantly in the SDM. This may be because the plant communities and soil properties only changed slightly and did not deteriorate before moderate degradation occurred, with the result that the habitat remained suitable for almost all soil nematode species. However, when the habitats severely deteriorated, the soil nematode diversity declined sharply because the physiology and activity of most invertebrates are adversely affected when certain environmental factors exceed their tolerance level^52^. The list of soil nematode genera in the four habitats (Table S1) also shows that many scarce genera that were present in other habitats disappeared from the SDM. The dynamics of abundance and diversity were only partially consistent with our first hypothesis that the abundance and diversity will decrease in response to alpine wetland degradation.

### Changes in the soil nematode trophic structure

The abundances of all trophic groups (except for predators) were significantly lower in the SDM than in the WM, and a similar phenomenon has previously been observed in a forest ecosystem^46^. Our regression analyses showed that the abundances of the five main trophic groups were positively correlated to plant species richness, soil N and water content. The decline of these environmental factors during degradation might explain the observed reduction in the abundances of the trophic groups. Previous studies have found that the effects of plant community and soil characteristics on soil nematodes are trophic group-specific^16,26,28,29,35^. The abundance of predators, although correlated with total N, did not change significantly between habitats, suggesting that the impacts of alpine wetland degradation do not extend to the higher levels of the soil food web. The organisms in the higher levels of the soil food web did not respond to changes in soil C^27^. This might result from the fact that predator species have a diverse prey preference and are thus not consistently limited by a single environmental factor. Regarding the changes in the relative abundance of each trophic group, significant differences between habitats were only recorded for omnivores and algivores. The regression analysis results also showed that only the relative abundances of the omnivores and algivores were significantly affected by plant coverage and available N, respectively. However, these two groups formed only a small percentage of the nematode community and thus contributed little to the overall pathway of energy flow through the nematode communities. These results indicate that while nematode abundances declined remarkably in response to wetland degradation, the relative abundances of most trophic groups remained stable. Therefore, we can infer that the main energy flow pathways through the nematode communities were only suppressed and not shifted during the process of wetland degradation. Consequently, our second hypothesis was not supported by our findings. In contrast, studies from other ecosystems have found that changes in soil properties^31,33^ and plant community^4,15^ alter the trophic structure of soil nematode communities. This may result from differences among ecosystem types.

### Seasonal dynamics and differences between habitats

The abundance, taxonomic richness and Pielou index varied significantly with sampling month, and the abundance and taxonomic richness were significantly affected by the interactions between habitat and sampling month. This may be attributable to seasonal changes in precipitation or temperature that occurred within our study area (Fig. 1). Previous studies have found that precipitation can increase nematode abundance^53^, and the taxonomic richness and Shannon index of soil nematodes depend on seasonal as well as short-term variations in temperature^34^. Additionally, the six trophic groups also responded differently to sampling month according to the temporal dynamics in their abundances and relative abundances. The predators, omnivores and algivores were more sensitive to sampling month than the herbivores, bacterivores and fungivores. This may result from the different influences of the plant community and soil property variables, which differed among the sampling months. A previous study showed that soil nematodes are affected by seasonal fluctuations in soil conditions^34^.

The seasonal dynamics in the community structure and diversity showed that the soil nematodes in the GM and MDM were more sensitive to sampling month than those in the WM and SDM. These findings suggest a close interaction between wetland degradation and seasonal fluctuations in plant community and soil properties in shaping soil nematode communities in alpine ecosystems. The reason behind this interaction may be that some environmental factors, e.g., the plant communities and soil properties, fluctuated more with season in the GM and MDM than in the other habitats. Other studies have shown that seasonal variations in climatic and soil factors can lead to changes in soil nematode communities^50,54^. In WM, the dominant plant species are perennial and hygrocolous, and the soil type is peat, which is less sensitive to temperature changes than the soils in the GM and MDM. Such differences indicate greater habitat stability in the WM than in the GM and MDM. At the extreme, the SDM, with sandy soils and lower plant coverage, has fewer water-filled pore spaces. Compared with more aggregated soils, soils in the SDM likely result in limited food resources for soil nematodes across all sampling months. This may partly explain the minimal temporal variation observed across the sampling months for the soil nematodes in the SDM.

## Conclusions

Our results show that the composition, abundance and diversity of the soil nematode communities in alpine wetlands have been significantly affected by climate- and land-use-driven degradation. The decreases in abundances of most nematode trophic groups showed that the main energy pathways through the soil nematode communities were suppressed by degradation; meanwhile, the changes in relative abundances showed that wetland degradation effects were fairly consistent across the most abundant nematode trophic groups, indicating no obvious shifts in the patterns of energy pathways through the soil nematode communities. The soil nematode communities in the original and severely degraded habitats were more stable across the seasons than were those in the intermediate degradation habitats, indicating that the stability of the soil nematode communities was closely related to the habitat stability. The relationships among soil nematode communities and the measured parameters of the plant community and soil properties suggested that changes in the plant community and soil properties will have important effects on soil nematodes during alpine wetland degradation.

## Electronic supplementary material

Supplemetary figure S1-4

Supplementary table S1
