# Author Correction: Impacts of alpine wetland degradation on the composition, diversity and trophic structure of soil nematodes on the Qinghai-Tibetan Plateau

**DOI:** 10.1038/s41598-018-23579-w

**Published:** 2018-04-05

**Authors:** Pengfei Wu, Hongzhi Zhang, Liwei Cui, Kyle Wickings, Shenglei Fu, Changting Wang

**Affiliations:** 1 College of Life Science and Technology, Southwest University for Nationalities, Chengdu, 610041 China; 2 Department of Entomology, New York State Agricultural and Experiment Station, Cornell University, Geneva, NY 14456 USA; 3 School of Environment and Planning, Henan University, Kaifeng, 475004 China

Correction to: *Scientific Reports* 10.1038/s41598-017-00805-5, published online 12 April 2017

This Article contains a typographical error in the Materials and Methods section under subheading ‘Data Analysis’.

$$H\text{'}=-{\sum }_{i=1}^{s}{p}_{i}{\rm{i}}\,\mathrm{ln}\,{p}_{i}$$

should read:

$$H\text{'}=-{\sum }_{i=1}^{s}{p}_{i}\,\mathrm{ln}\,{p}_{i}$$

