# Supplementary material for: Impacts of alpine wetland degradation on the composition, diversity and trophic structure of soil nematodes on the Qinghai-Tibetan Plateau
Source: Sci Rep. 2017 Apr 12;7:837. doi: 10.1038/s41598-017-00805-5 (PMC5429801; doi:10.1038/s41598-017-00805-5)
Supplement: Supplementary file 1 — Supplemetary figure S1-4 [file 41598_2017_805_MOESM1_ESM.doc]

Supplemetary information

Impacts of alpine wetland degradation on the composition, diversity and trophic structure of soil nematodes on the Qinghai-Tibetan Plateau

Pengfei Wu^1*^, Hongzhi Zhang^1^, Liwei Cui^1^, Kyle Wickings^2^, Shenglei Fu^3^, Changting Wang^1^

1 College of Life Science and Technology, Southwest University for Nationalities, Chengdu 610041, China

2 Department of Entomology, New York State Agricultural and Experiment Station, Cornell University, Geneva, NY 14456, USA

3 School of Environment and Planning, Henan University, Kaifeng 475004, China

*Corresponding author: wupf@swun.cn


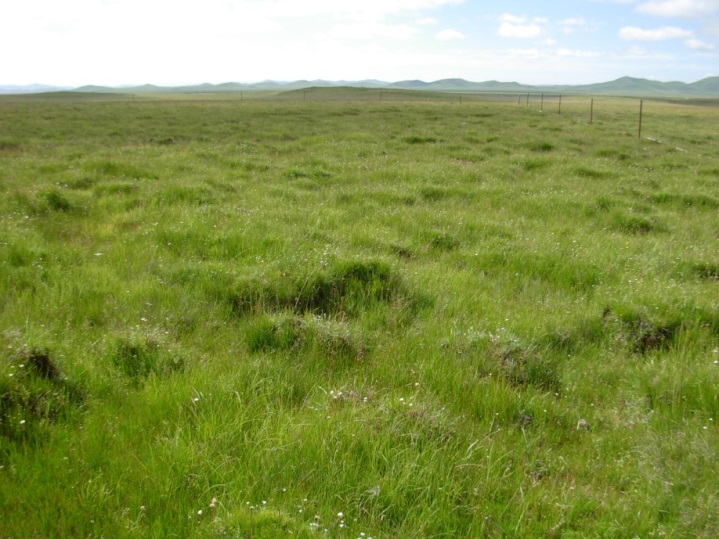


Figure S1 Wet meadow

Habitat in the figure is wet meadow in our manuscript, also called as drought marsh in the Zoigȇ wetland. The dominant species in this community are *Elymus nutans, Ranunculus tanguticus, Leontopodium nanum, Polygonum viviparum, Taraxacum maurocarpum, Aster tataricus, Trollius ranunculoides* and *Geranium pylzovianum*. The height of plants ranges from 10 cm to 30 cm, and the plant coverage is more than 90%. The soil of the wet meadow is peat soil. The swampy meadow distributes widely in the Zoigȇ wetland.


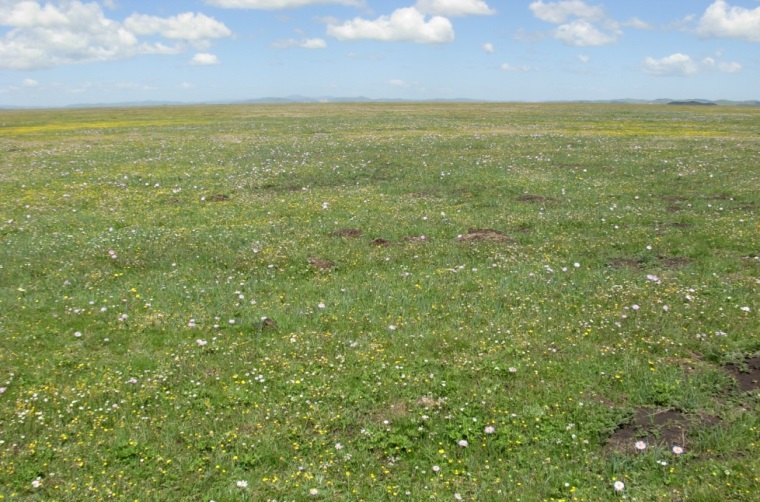


Figure S2 Grassland meadow

Habitat in the figure is grassland meadow in our manuscript, which develops from wet meadow because of water running off. The most common species are *Festuca ovina, Kobresia setchwanensis, Ranunculus tanguticus, Carum carvi, Stellera chamaejasme, Anaphalis lacteal, Anemone trullifolia, Trollius farreri, Elymus nutans, Poa pratensis,* and *Oxytropis Kansuensis*. The height of plants ranges from 20 cm to 40 cm and the plant coverage is more than 90%. The soil of the grassland meadow is sandy loam. The grassland meadow is also distributes widely in the Zoigȇ wetland.


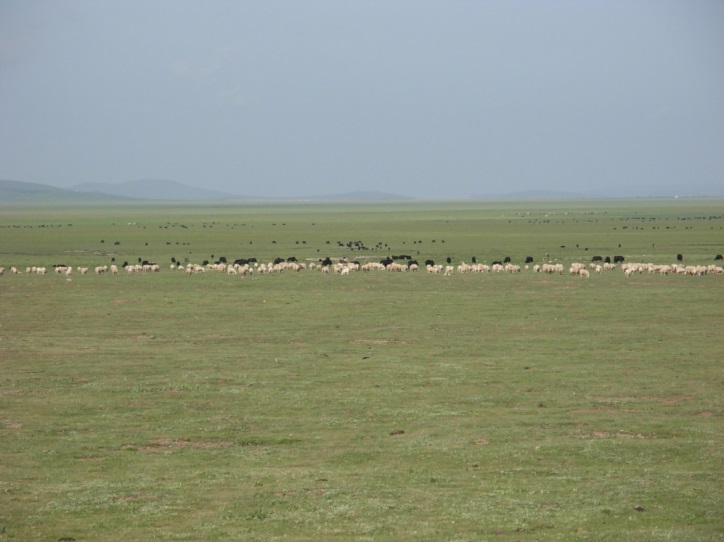


Figure S3 Moderately degraded meadow

Habitat in the figure is the moderately degraded meadow in our manuscript, which develops from grassland meadow because of effects of climate change and heavy grazing. The dominant species in this community are *Potentilla ansrina, Kobresia bellardii, Ligularia virgaurea, Taraxacum lugubre Dahlst, Ranunculus tanguticus,* and *Oxytropis Kansuensis*. The height of plants ranges from 0 cm to 5 cm, and the plant coverage is approximately 50%-70%. The soil of the moderately degraded meadow is sandy loam. The area of moderately degraded meadow is increasing in the Zoigȇ wetland.


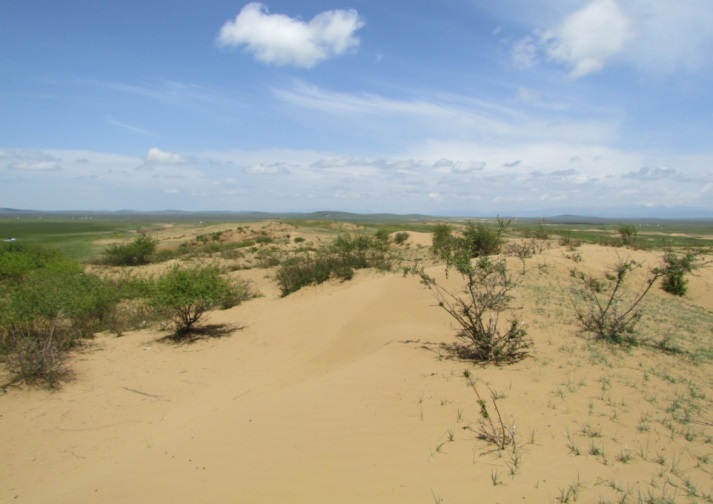

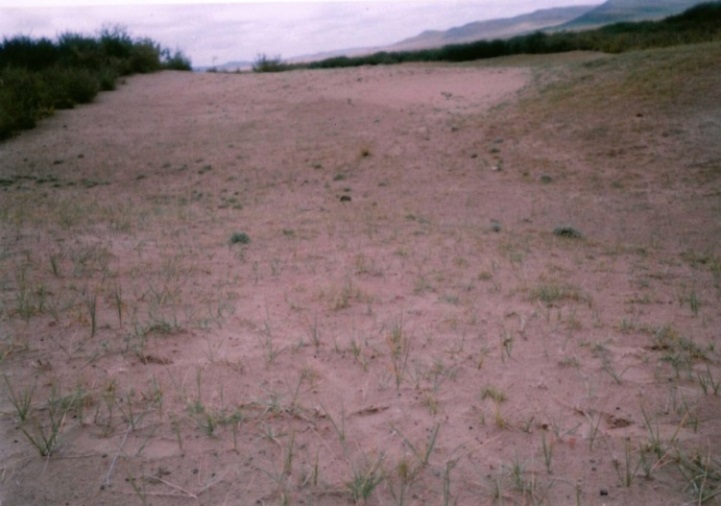


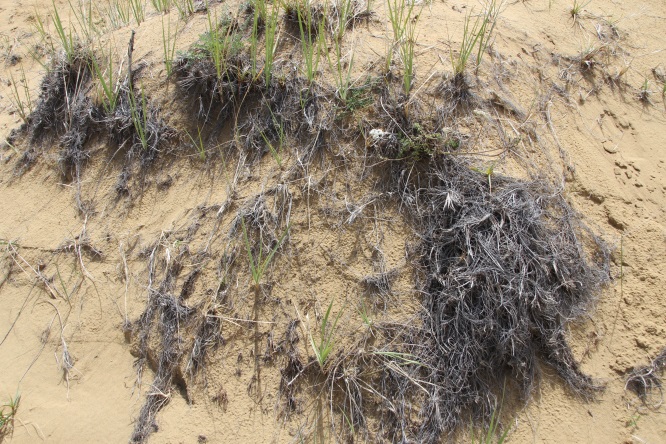

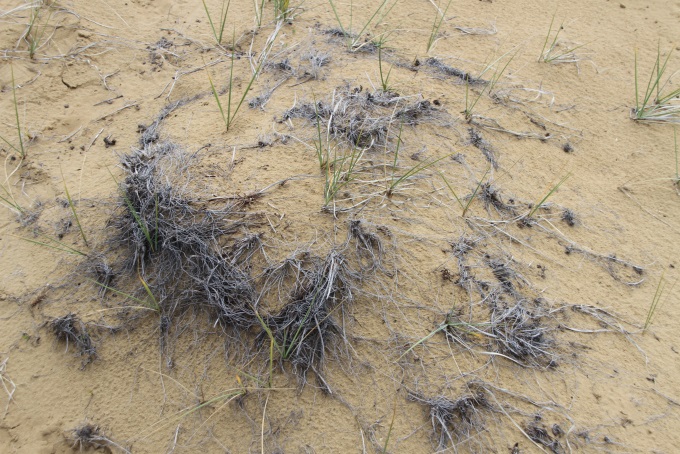


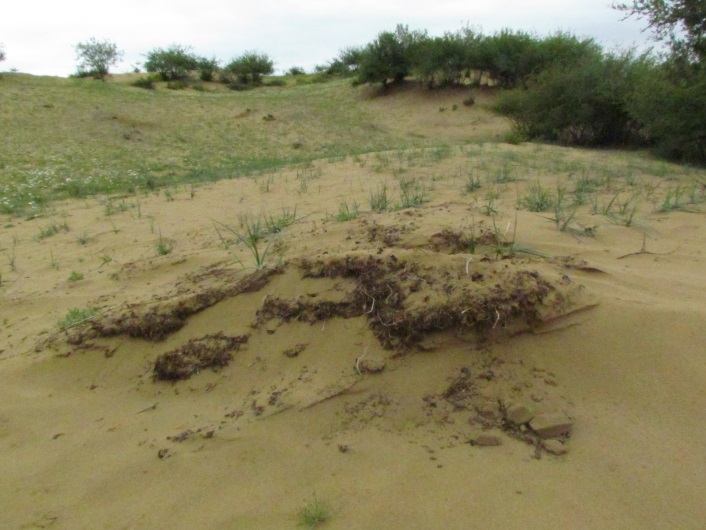

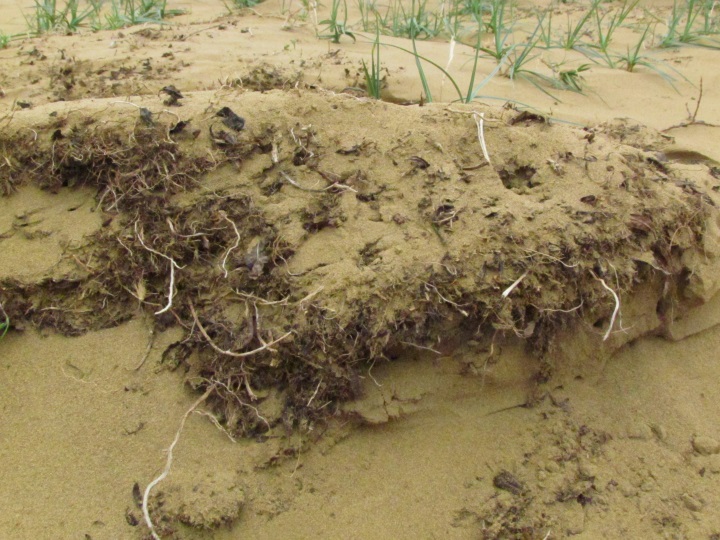


Figure S4 Severely degraded meadow

Habitat in the figure is severely degraded meadow in our manuscript, which develops from the moderately degraded meadow because of climate change and heavy grazing. The dominant plant species in this community are *Elymus nutans and Carum carvi*. The plant height ranges from 0 cm to 20 cm and the plant coverage is less than 10%. The soil of the severely degraded meadow is sandy soil. The area of severely degraded meadow is stable in recent years because of many measures was used to prevent the grassland from desertification in the Zoigȇ wetland.
